# Law abidance leadership education for university students in Hong Kong: Post-lecture evaluation

**DOI:** 10.3389/fpsyg.2022.994448

**Published:** 2022-10-28

**Authors:** Daniel T. L. Shek, Diya Dou, Xiaoqin Zhu, Xiang Li

**Affiliations:** Department of Applied Social Sciences, The Hong Kong Polytechnic University, Hung Hom, Hong Kong SAR, China

**Keywords:** law abidance, leadership, subjective outcome evaluation, national security, Hong Kong National Security Law

## Abstract

Law abidance is very important for effective leaders. Without law abidance, abuse of power and corruption would easily happen, which would eventually erode organizational health. To promote law abidance leadership in university students in Hong Kong, we developed a law abidance leadership program with 3 h of face-to-face lecture and 7 h of self-study of materials disturbed to students. To understand students’ perception of the 3-h lecture, we conducted a post-lecture evaluation study using a 26-item measure (*N* = 914). Results showed that the scale possessed good reliability and validity. Confirmatory factor analyses showed that the assessment tool has three internally consistent and factorial invariant dimensions: program attributes, appreciation of law abidance, and teacher attributes. Regarding students’ perception of the program, students were generally satisfied with the lecture attributes, including design, content, lecture atmosphere, teaching quality, and benefits to students. In particular, students agreed that the lecture helped them understand the importance and value of law abidance and national security; over 95% of the students indicated that they would try their best to serve as law-abiding citizens and socially responsible leaders. Multiple regression analyses showed that program attributes, appreciation of law abidance, and teacher attributes predicted overall satisfaction with the lecture. Qualitative comments of the student echoed the quantitative findings, with most of the comments being positive in nature. The present study replicated the findings reported previously. Local and international contextual factors relevant to the interpretations of the findings are highlighted.

## Introduction

Successful leadership has many attributes, such as effective communication and problem-solving skills (Shek et al., 2018). In particular, successful leaders should follow the rules and regulations within the organizations and the society because inability and/or unwillingness to follow the law will result in organizational corruption and inefficiency. In the past decades, top political leaders in Korea, Taiwan, and Hong Kong were imprisoned because of corruption. These cases clearly illustrate that law abidance is an important foundation of effective leadership in organizational and political contexts. As young people are leaders of tomorrow, leadership training is important for young people, particularly in the area of law abidance (Shek et al., 2022b). This focus is particularly important because conduct problems such as cheating and plagiarism are not uncommon in young people. There are studies showing that cheating is widespread (Owunwanne et al., 2010; Anitha and Sundaram, 2021), and academic dishonesty is a growing problem in different societies (Chen and Chou, 2017).

There are several explanations of non-law-abidance in young people. First, young people may lack the knowledge and understanding of the importance of obeying the law. In other words, “ignorance” due to an inadequate understanding of the requirement of the law is a possible contributing factor. A lack of cognitive competence may also contribute to the lack of law abidance. For example, information literacy, as an aspect of cognitive competence, refers to the ability to critically think, evaluate, synthesize, organize, and communicate information (Andretta, 2005). Researchers have found that strong information literacy helps people be aware of personal biases and identify fake political news and misinformation regarding social issues (Jones-Jang et al., 2021). Second, young people do not obey the law because of “adolescent rebellion.” Young people disobey the law because they can practice their sense of autonomy and validate their identity (Pozzi et al., 2018). Third, based on the General Strain Theory (Agnew, 1992), delinquent disobedience of laws is regarded as a coping to release individuals’ stress and tension (Xu et al., 2021; Wang et al., 2022). As Hong Kong is a highly competitive city where young people have to endure huge stress such as career challenges, high property prices, and low income (Shek and Siu, 2019), psychological distress is prevalent in young people (Li et al., 2021). To relieve stress, some young people may commit delinquent acts or disobey the law. Fourth, Social Learning Theory (Bandura, 1977) can be used to explain why some people lack the willingness to follow the law. In the social learning process, individuals learn to disobey the law by observing and imitating people who are similar or superior to them (Juwel and Ahsan, 2019). Finally, Differential Association Theory (Sutherland, 1973) has been used to explain the learning process of criminal behaviors (Kolthoff, 2020). Young people can learn criminal behaviors through their interaction and communication with intimate groups (Antwi Bosiakoh, 2012). In these explanations, one common element is that young people and their peer group lack proper legal knowledge and awareness of the importance of law abidance, hence making them easily influenced by their peers and prone to disobey the law to release their stress. In fact, there are findings showing that Hong Kong young people did not have a good understanding of law abidance and they tended to disobey the law if they feel that the law is “unreasonable” (The Hong Kong Federation of Youth Groups, 2021). Hence, helping young people to understand laws of the place and develop respect for law abidance are important tasks for educators.

With specific reference to Hong Kong, there are two major “social events” in the past decade. In 2014, the “Occupy Central Movement” (or “Umbrella Movement”) took place in Hong Kong. The protestors (including many young people) occupied the major roads in Hong Kong for 79 days, which seriously affected the lives of many Hong Kong people. Nevertheless, the movement was basically peaceful without much violence. In contrast, in 2019–2020, the “Social Event” in response to the Anti-Extradition Law Amendment Bill Movement took place in Hong Kong (Shek, 2020). Although many demonstrations were initially peaceful, they ended up with widespread vandalism (such as damaging station facilities of Mass Transit Railway), violence (such as beating people holding different political views), and disruption of public order (such as occupying university premises and blocking main roads) which lasted for over a year up to June 2020.

Because of such developments, the Standing Committee of the National People’s Congress passed “The Law of the People’s Republic of China on Safeguarding National Security in the Hong Kong Special Administrative Region” (also known as the “Hong Kong National Security Law” or NSL) based on the “Decision of the National People’s Congress on Establishing and Improving the Legal System and Enforcement Mechanisms for the Hong Kong Special Administrative Region to Safeguard National Security” of the National People’s Congress. Because of social instability and violence in 2019, many people welcomed the introduction of the “Hong Kong National Security Law.” Actually, restoring social order did not only attributed to the lockdown and quarantine restrictions under the COVID-19, but also to the introduction of NSL. Besides, national security laws are commonly enacted in different parts of the world. On the other hand, as the Hong Kong National Security Law was enacted by the National People’s Congress, some people might have the feeling that such a “top-down” initiative would unreasonably restrict the rights and freedom of Hong Kong people. In particular, there are skeptical views suggesting that NSL is a “legitimate” instrument for social control and silencing criticisms of the Hong Kong Government and the Central Government.

As there are different views on the NSL, a proper and correct understanding of the Hong Kong National Security Law is indispensable to promote public acceptance. In fact, in Article 10 of the Hong Kong National Security Law, it is stated that “The Hong Kong Special Administrative Region shall promote national security education in schools and universities and through social organizations, the media, the internet and other means to raise the awareness of Hong Kong residents of national security and of the obligation to abide by the law.” In other words, it is the responsibility of universities to educate students on the Hong Kong National Security Law (i.e., universities cannot say “no”). Because of this requirement, there is a need to understand the experience of the students receiving NSL education. The rationale for evaluating NSL education is simple–if students are not satisfied with the NSL education, they will resent obeying the related law, and the “law” behind NSL cannot be realized.

To promote NSL education for undergraduate students, we designed a NSL program with 3 h of face-to-face lectusre and 7 h of self-study in a public university in Hong Kong. For the 3-h lecture, we covered law abidance leadership (e.g., the reasons why a leader should obey law), a brief overview of modern Chinese history (“Century of Humiliation”), the concept of national security, the Hong Kong National Security Law, and issues surrounding the Hong Kong National Security Law. Shek et al. (2022b) presented evaluation findings on the first implementation of the 3-h law abidance leadership lecture conducted in the first semester of 2021/22 academic year. With reference to the quantitative findings, results showed that students generally had positive reactions to the NSL lecture, including the lecture (design, content, and process), teacher, benefits, and law abidance leadership. With specific reference to law abidance, 91 and 92% of the respondents agreed that the lecture helped them understand the concept of national security and offenses in the Hong Kong National Security Law, respectively. In particular, 92% of the respondents agreed that “I will try my best to serve as a socially responsible leader.” Similarly, the qualitative responses are basically positive in nature, which echoed the quantitative findings based on the survey responses. Some responses included “I felt happy to hear different points of view from classmates on NSL” and “I have learned different types of crimes and I did not know that a lot of countries have implemented NSL. Through this lecture, I have learned and gained deeper knowledge about laws.”

Although responses of the students in our first attempt appeared to be positive, there is a need to replicate the findings across time, particularly under COVID-19. During the pandemic, there is a need to conduct different modes of teaching, including online mode and hybrid mode. In the study conducted by Shek et al. (2022b), we used a hybrid teaching mode (i.e., face-to-face class plus online teaching) to conduct the 3-h lecture.

During the pandemic, there is a practical need to transform teaching and learning because of social distancing measures (Rashid and Yadav, 2020; Kohnke and Moorhouse, 2021). In the scientific literature, there are different findings on the impact of online learning on student satisfaction. On the one hand, some studies underscore the benefits of online learning, such as the promotion of self-autonomy in the learning process (Gonzalez et al., 2020). Online learning activities, such as polling, word cloud, and Padlet, can protect student anonymity (Barr, 2017), which may promote their active engagement in a course about law abidance leadership and NSL. On the other hand, researchers also identified problems of online learning, such as a lack of preparation and readiness to learn (Nassr et al., 2020). Students who have a lower motivation to study NSL may be less engaged in online learning as compared to a face-to-face mode. As such, there is a need to understand the difference in students’ reactions to in-person versus online teaching mode affecting their satisfaction with NSL education.

In this paper, we focus on student responses to the 3-h lecture in Semester 2 of 2021/22 academic year *via* post-lecture evaluation (26-item measure plus open-ended questions). As Hong Kong experienced the fifth wave of COVID-19 in Hong Kong since early January 2022, the lecture was conducted *via* online mode without face-to-face teaching. With reference to existing measures of student satisfaction (Shek and Sun, 2014; Sun and Shek, 2014), we developed a 26-item measure to assess students’ perceptions of NSL education. Conceptually, we included items assessing perceptions of three domains, including the perceived lecture attributes, teacher attributes and appreciation of law abidance.

Subjective outcome evaluation based on a client satisfaction approach has a long history in the evaluation field, including higher education (Fraser and Wu, 2016; Sozer et al., 2019; Zhu et al., 2021). Several advantages of using this evaluation strategy in education are noted. First, it can generate timely feedback as in the case of post-lecture evaluation. Second, it is easy and inexpensive for front-line teachers to collect data without requiring advanced skills or equipment. Third, accumulated information and data on client satisfaction from different student samples can reveal a reliable figure on how the course is perceived by students (Shek and Sun, 2014; Lin et al., 2022; Shek et al., 2022a). However, it has also been criticized for its subjective nature. In particular, favorable findings obtained from subjective outcome evaluation do not necessarily mean favorable changes in students. Nevertheless, this criticism can be overcome by the utilization of validated and reliable assessment tools. Indeed, if the utilized assessment tool is valid and reliable, findings from subjective outcome evaluation are well-correlated with student changes after receiving the course (Shek, 2014; Sun and Richardson, 2016; Zhu et al., 2021).

In this study, we explored the following research questions based on the perspective of the students:

Research Question 1: What are the psychometric properties of the 26-item measure of the subjective perceptions of the students of the 3-h lecture on law abidance leadership? With reference to the conceptual model that the measure covers lecture attributes, teacher attributes, and appreciation of law abidance, we expected that there would be support for the 3-factor model of the subjective outcome evaluation tool (Hypothesis 1).

Research Question 2: How do students perceive the 3-h law abidance leadership lecture regarding the lecture attributes (design, content, process, and benefits), teacher’s attributes, and appreciation of law abidance leadership? With reference to Shek et al. (2022b), we expected that most students would have positive perceptions of different aspects of the lecture (Hypothesis 2).

Research Question 3: What are the predictors of overall satisfaction with the lecture? Based on the previous studies (Shek and Sun, 2014; Sun and Shek, 2014), we expected that perceived lecture attributes, teacher attributes, and appreciation of law abidance leadership would predict overall satisfaction with the lecture (Hypothesis 3a–3c).

Research Question 4: Based on the qualitative findings, what are students’ perceptions of the 3-h lecture on law abidance leadership? With reference to the observations of Shek et al. (2022b), we expected that the profile of qualitative responses would be basically positive, with fewer would be more positive responses than negative responses (Hypothesis 4).

## Materials and methods

### Participants and procedures

In this study, we focused on students’ perceptions after attending one lecture on law abidance leadership, including perceived lecture attributes, teachers’ attributes, and appreciation of law abidance leadership. At the largest public university in Hong Kong, “Tomorrow’s Leaders” is a credit-bearing leadership subject offered to all year 1 students from different faculties and schools, except the Faculty of Business which has its own leadership subject. It consists of 13 lessons, with a 3-h lecture each week. This subject is developed to nurture young students’ leadership competencies and their intra- and inter-personal competencies, such as cognitive competence, social competence, moral competence, communication, conflict management, and law abidance leadership. This subject has received international recognition and affirmation, including the Silver Award (Ethical Leadership) and Gold Award (Nurturing Student Well-Being and Purpose) in QS Reimagine Education Awards in 2017 and 2021, respectively. The subject was also awarded the University Grants Committee Teaching Award in Hong Kong in 2018, which is the most prestigious teaching award in the higher education sector in Hong Kong.

For the lecture on law abidance leadership, we cover several topics, including law abidance leadership, a brief overview of modern Chinese history (“Century of Humiliation”), the concept of national security, and the Hong Kong National Security Law, including its major offenses and related issues. In addition to the 3-h lecture, we also distributed 60 lecture notes to the students covering different areas, including a brief overview of the history of modern China, “Century of Humiliation,” resuming exercise of sovereignty over Hong Kong by the Chinese Government in 1997, the Constitution of China, the concept of national security, and the Hong Kong National Security Law.

After attending the 3-h lecture on law abidance leadership, all students were invited to reflect on and evaluate their learning experiences in terms of perceptions on lecture attributes, teacher attributes and appreciation of law abidance in a post-lecture evaluation survey. A total of 914 students (around 70% out of 1,308 registered students) completed the survey. The students were from seven faculties or schools, including the Faculty of Construction and Environment, Faculty of Engineering, Faculty of Health and Social Sciences, Faculty of Science, School of Design, School of Fashion and Textiles, and School of Hotel and Tourism Management. To minimize the sensitivity involved in participation, demographic data were not collected. According to the University’s figures, the mean age of the students was around 18 years. We had obtained institutional ethics approval before we launched the study.

### Instruments

Based on a study conducted by Shek et al. (2022b), we used the following items in different areas in this study:

#### Perception of the lecture attributes

- “The design of this lecture was very good.” (Item 1)
- “The classroom atmosphere of this lecture was very pleasant.” (Item 2)
- “There was much peer interaction amongst the students in this lecture.” (Item 3)
- “There was much interaction between the lecturer and the students in this lecture.” (Item 4)
- “There was much student participation in this lecture.” (Item 5)
- “There were many opportunities for reflection in this lecture.” (Item 6)
- “This lecture is helpful to my personal development.” (Item 7)
- “This lecture has improved my problem-solving ability.” (Item 8)
- “This lecture has improved my understanding of the importance of attributes of successful leaders (e.g., critical thinking, moral competence, and law abidance).” (Item 9)
- “This lecture has improved my interpersonal communication skills.” (Item 10)
- “This lecture has improved my critical thinking.” (Item 11)

#### Appreciation of law abidance leadership

- “This lecture helps me understand the importance of law abidance in leadership.” (Item 12)
- “I understand that law abidance is important for the stability of a society.” (Item 13)
- “This lecture helps me understand the concepts of national security.” (Item 14)
- “This lecture helps me understand the offenses and penalties surrounding the National Security Law in Hong Kong.” (Item 15)
- “This lecture helps me understand the importance of implementing the National Security Law in Hong Kong.” (Item 16)
- “This lecture helps me clarify some myths related to National Security Law in Hong Kong.” (Item 17)
- “I will try my best to serve as a law-abiding citizen.” (Item 18)
- “I will try my best to serve as a socially responsible leader.” (Item 19)

#### Teacher’s attributes

- “The lecturer had a good mastery of the lecture material.” (Item 21)
- “The lecturer used different methods to encourage students to learn.” (Item 22)
- “The lecturer was able to promote an atmosphere of mutual respect in the class.” (Item 23)
- “The lecturer was able to help students understand the knowledge covered in the lecture.” (Item 24)
- “The lecturer was able to effectively take care of all students.” (Item 25)
- “Overall speaking, I have a very positive evaluation of the lecturer in this lecture.” (Item 26)

#### Overall evaluation

- “Overall speaking, I have a very positive evaluation of this lecture.” (Item 20)

## Results

Table 1 summarizes the means, SD, and inter-correlations among the three subscales (i.e., perception of the lecture attributes, appreciation of law abidance leadership, and teacher’s attributes). Significant inter-correlations were found between all subscales (*r*s > 0.84). Table 2 summarizes the results of the normality test, reliability, and validity. The values of skewness and kurtosis of all items in the scale were lower than 2 and 7, respectively, and thus met the requirement of normality recommended by Curran et al. (1996). According to Fornell and Larcker (1981), we used average variance extracted (AVE) and composite reliability (CR) to establish convergent validity, with cutoffs of 0.50 and 0.70, respectively (Fornell and Larcker, 1981). As shown in Table 2, the AVE values and CR for the three subscales were larger than 0.50 and 0.70, respectively, suggesting that the scale had a good convergent validity. We evaluated the discriminant validity by comparing the square root of the AVE of each factor with the correlation coefficients. The correlation coefficients between factors ranged between 0.841 and 0.888, which were smaller than the square root of the AVE for each factor (ranging between 0.898 and 0.938), and thus supported the discriminant validity of the scale. As for the reliability, all subscales and the full scale showed good reliability with Cronbach’s alphas larger than 0.97 and McDonald’s omegas greater than 0.97.

**TABLE 1:** Descriptive statistics and correlations among sub-scale.

		Mean	SD	1	2
1	Perception of the lecture attributes	4.542	1.021		
2	Appreciation of law abidance leadership	4.671	0.993	0.888***	
3	Teachers’ attributes	4.807	0.892	0.841***	0.855***

***p < 0.001.

**TABLE 2:** Reliability and validity of full scale and factors of the scale.

	Skewness	Kurtosis	Factor loading	Cronbach’s alpha	McDonald’s omega	Mean inter-item correlation	AVE	CR
Factor 1: Perception of the lecture attributes (11 items)				0.979	0.980	0.813	0.807	0.979
Item 1	–0.95	1.51	0.904					
Item 2	–0.87	1.12	0.896					
Item 3	–0.77	0.93	0.819					
Item 4	–0.89	1.69	0.848					
Item 5	–0.89	1.25	0.856					
Item 6	–0.91	1.26	0.935					
Item 7	–0.99	1.11	0.942					
Item 8	–0.88	0.93	0.932					
Item 9	–1.04	1.54	0.927					
Item 10	–0.81	0.83	0.907					
Item 11	–1.01	1.45	0.904					
Factor 2: Appreciation of law abidance leadership (8 items)				0.973	0.974	0.821	0.821	0.973
Item 12	–1.05	1.79	0.943					
Item 13	–1.05	2.11	0.927					
Item 14	–1.14	2.11	0.922					
Item 15	–1.04	1.90	0.940					
Item 16	–1.07	1.40	0.929					
Item 17	–0.98	1.33	0.920					
Item 18	–1.14	2.62	0.828					
Item 19	–1.06	2.39	0.831					
Factor 3: Teacher’s attributes (6 items)				0.978	0.978	0.881	0.879	0.978
Item 21	–0.99	2.27	0.917					
Item 22	–0.92	2.13	0.944					
Item 23	–0.97	2.35	0.949					
Item 24	–0.97	2.25	0.958					
Item 25	–0.99	2.23	0.929					
Item 26	–1.08	2.47	0.929					
Full scale				0.988	0.989	0.774		

**FIGURE 1:**
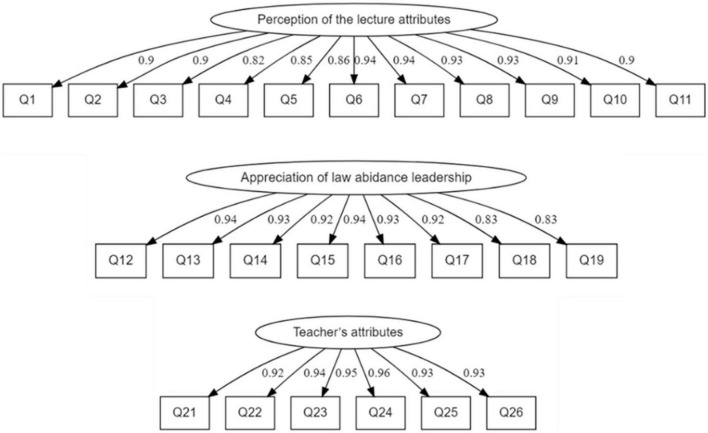
The CFA model.

We used structural equation modeling (SEM) to examine the validity and dimensionality of the scales, which is a commonly used statistical technique in the field of assessment (Kline, 2015). SEM model allows for the simultaneous estimation of observed variables, latent variables, and the relationships between them. It also verifies whether the estimated factors are measuring the same underlying latent construct among groups or across time points. Researchers have suggested that robust maximum likelihood (MLR) is appropriate for Likert-scales with five or more response categories (Byrne, 2012). We adopted MLR estimation for confirmatory factor analysis (CFA) using lavaan package in R software (Rosseel, 2012). We first performed CFA on the three-factor model with the entire sample. The results showed that while some model fit indices were acceptable, some were less satisfactory. Further examination of the modification indices showed that there were 13 pairs of items with high modification indices. These pairs of items either shared content (e.g., both item 3 and item 4 focus on interactions in class) or formulated the statements in a similar format (e.g., item 18 and item 19 start with “I will try my best to serve as a…”). Hence, we revised the model by setting the residual covariance for each pair of items. The revised model showed improved model fit indices [χ^2^(259) = 1526.521, CFI = 0.973, NNFI = 0.968, RMSEA = 0.065, SRMR = 0.029]. Thus, Hypothesis 1 is supported. The model is graphically presented in Figure 1.

Before conducting multi-group CFA, we tested the baseline model in subsamples to gauge the factorial stability across subsamples (Byrne, 2012). The baseline model demonstrated a good fit to the data in both subsample A and subsample B (CFI > 0.96, NNFI > 0.96, RMSEA ≤ 0.072, SRMR ≤ 0.038, see Table 3). Next, we tested measurement invariance across the subsamples by adding constraints into a series of nested models. First, Model 1 (configural invariance model) did not include equality constraints. As shown in Table 3, the model fit indices of Model 1 were in line with recommended benchmarks, yielding configural invariance across subsamples. Second, we constrained factor loadings to be equal across subsamples in Model 2 (weak invariance model), which also fitted the data well. Comparison between Model 2 and Model 1 yielded an absolute value of ΔCFI (-0.001) below 0.01 and ΔRMSEA (0.001) below 0.015, suggesting invariance in factor loadings across subsamples. In Model 3 (strong invariance model), both factor loadings and measurement intercepts were assumed to be equal across subsamples. Model comparison between Model 3 and Model 2 revealed an unchanged CFI and an increase of 0.001 in RMSEA, which stayed below the recommended benchmarks, and thereby supported invariance in factor loadings and intercepts across subsamples. Lastly, we constrained residuals to be equal across subsamples in Model 4 (strict invariance model). The values of ΔCFI (0.001) and ΔRMSEA (0.003) were below 0.01 and 0.015, respectively, denoting the invariance of measurement error for each item across subsamples.

**TABLE 3:** Summary of goodness-of-fit for CFA and invariance tests.

Model no.	Model description	χ ^2^	Δχ ^2^	*df*	Δ *df*	CFI	Δ CFI	NNFI	RMSEA	RMSEA 95% CI		Δ RMSEA	SRMR
										Lower	Upper		
0	Baseline model	1526.521	–	259	–	0.973	−	0.968	0.065	0.059	0.072	–	0.029
0a	Baseline model (Subsample A)	1126.929	–	259	–	0.969	−	0.964	0.072	0.063	0.081	–	0.029
0b	Baseline model (Subsample B)	1089.278	–	259	–	0.968	−	0.962	0.069	0.060	0.079	–	0.038
1	Configural invariance	2216.206	–	518	–	0.968	−	0.963	0.071	0.064	0.077	–	0.032
2	Metric invariance	2270.051	53.845	540	22	0.967	–0.001	0.964	0.070	0.063	0.076	0.001	0.040
3	Scalar invariance	2289.429	19.378	562	22	0.967	0.000	0.965	0.068	0.062	0.075	0.001	0.040
4	Error variance invariance	2377.043	87.614	587	25	0.968	0.001	0.968	0.066	0.060	0.073	0.003	0.039

N_whole_ = 914; N_subsampleA_ = 457; N_subsampleB_ = 457; CFI, Comparative Fit Index; RMSEA, Root Mean Square Error of Approximation; CI, confidence interval; Δχ^2^, change in χ^2^ compared to the previous model; Δdf, change in degrees of freedom compared to the previous model; ΔCFI, change in CFI compared to the previous model; Model 0, Baseline model using the whole sample; Model 0a, Baseline model using subsample A; Model 0b, Baseline model using subsample B; Model 1, no equality constraints were imposed; Model 2, equality constraints were imposed on all factor loadings; Model 3, equality constraints were imposed on all factor loadings and intercepts of the measured variables; Model 4, equality constraints were imposed on all factor loadings, intercepts, and residual variance.

Table 4 presents the percentages of responses to the items in the scale. There are several observations based on the findings. First, the students held positive views about the design and delivery of the lecture: 90% viewed that the lecture was well-deigned and 89% and 89% agreed that there was much student participation and the lecture enabled them to have reflection, respectively. Besides, students perceived that the lecture benefitted their personal development (87%), problem-solving ability (86%), knowledge of the attributes of successful leaders (90%), interpersonal communication skills (86%), and critical thinking (89%). Regarding appreciation of law abidance, results showed that most of the students appreciated the lecture: 92% and 92% of the students agreed that the lecture helped them understand “the importance of law abidance in leadership” and the “concepts of national security,” respectively; 88.5% of the students perceived that the lecture clarified myths surrounding the Hong Kong National Security Law. Most importantly, 95% and 95% of the students agreed that they would try their best to “serve as a law-abiding citizen” and “serve as a socially responsible leader,” respectively.

**TABLE 4:** Responses of the students to the post-lecture evaluation questionnaire (*N* = 914).

Questionnaire item		*Mean*	Positive response rate%
1	The design of this lecture was very good.	4.57	89.53%
2	The classroom atmosphere of this lecture was very pleasant.	4.52	87.33%
3	There was much peer interaction amongst the students in this lecture.	4.54	87.54%
4	There was much interaction between the lecturer and the students in this lecture.	4.70	92.72%
5	There was much student participation in this lecture.	4.59	88.74%
6	There were many opportunities for reflection in this lecture.	4.56	88.89%
7	This lecture is helpful to my personal development.	4.48	86.56%
8	This lecture has improved my problem-solving ability.	4.45	85.57%
9	This lecture has improved my understanding of the importance of attributes of successful leaders (e.g., critical thinking, moral competence, law abidance, etc.).	4.58	89.88%
10	This lecture has improved my interpersonal communication skills.	4.43	85.59%
11	This lecture has improved my critical thinking.	4.56	89.34%
12	This lecture helps me understand the importance of law abidance in leadership.	4.65	91.64%
13	I understand that law abidance is important for the stability of a society.	4.68	93.16%
14	This lecture helps me understand the concepts of national security.	4.61	91.62%
15	This lecture helps me understand the offenses and penalties surrounding the National Security Law in Hong Kong.	4.70	92.60%
16	This lecture helps me understand the importance of implementing the National Security Law in Hong Kong.	4.55	88.44%
17	This lecture helps me clarify some myths related to National Security Law in Hong Kong.	4.58	88.48%
18	I will try my best to serve as a law-abiding citizen.	4.81	95.36%
19	I will try my best to serve as a socially responsible leader.	4.79	95.37%
20	Overall speaking, I have a very positive evaluation of this lecture.	4.59	89.08%
21	The lecturer had a good mastery of the lecture material.	4.81	95.24%
22	The lecturer used different methods to encourage students to learn.	4.80	95.25%
23	The lecturer was able to promote an atmosphere of mutual respect in the class.	4.83	95.93%
24	The lecturer was able to help students understand the knowledge covered in the lecture.	4.79	95.03%
25	The lecturer was able to effectively take care of all students.	4.79	94.91%
26	Overall speaking, I have a very positive evaluation of the lecturer in this lecture.	4.82	95.26%

All items were rated on a 6-point Likert scale (1 = Strongly Disagree; 2 = Disagree; 3 = Slightly Disagree; 4 = Slightly Agree; 5 = Agree; 6 = Strongly Agree).

Finally, consistent with our expectation, students generally showed positive evaluation of the attributes of teachers, including their mastery of lecture materials (95%), encouragement of students to learn *via* different methods (95%), creation of mutual respect in class (96%), and able to help students to understand the subject matter (95%). Taken as a whole, the findings provided support to the expectation that students showed positive evaluation of the lecture and teachers, as well as an appreciation of law abidance leadership. The findings provide support to Hypothesis 2.

Regarding the predictors of overall satisfaction with the lecture, multiple regression analysis with lecture attributes, teacher attributes, and appreciation of law abidance as predictors and overall satisfaction with the lecture as the criterion variable was performed. In line with our predictions, lecture attributes (beta = 0.272, *p* < 0.001), teacher attributes (beta = 0.069, *p* < 0.05), and appreciation of law abidance (beta = 0.604, *p* < 0.001) were significant predictors of overall satisfaction with the lecture. These findings give support to Hypotheses 3a, 3b, and 3c.

Finally, an examination of the qualitative comments of the students in Table 5 showed several observations. First, the number of positive responses outnumbered the negative, neutral and unclassified responses. Second, students had positive views about the lecture attributes (such as positive attributes of the lecture), teacher attributes (such as appreciation of the teachers) and appreciation of law abidance (such as acquiring knowledge about the Hong Kong National Security Law). Third, students generally had positive views about national security and the Hong Kong National Security Law. Generally speaking, the qualitative picture based on the students who responded to the questionnaire was generally positive, which gives support to Hypothesis 4.

**TABLE 5:** Comments of the students on the lecture on law abidance leadership (*N* = 85) with examples given under each category or sub-categories.

Positive responses (109 responses)	
“General Positive Comments” Category (5 responses)	#Good. #Good. #Great. #Good lecture. # it is a good experience to let us have this lesson in this course.
“Appreciation of the Lecture and Related Materials” Category (18 responses)	#The 60 Lecture Notes is a good summary of ideas. #The Lecture Notes contain some well-organized content. #Like this lecture a lot. #Really enjoy it! #Friendly. #Active. #Fun. #Interesting. #Engaging. #The concepts are clearly delivered. #This lecture is well organized. #The lesson is interesting. #We can engage in the lesson easily. #Class exercises are engaging. #Clear presentation. #It is a meaningful lesson. #This lecture is very informative. #Interactive.
“Useful/Helpful/Gain Knowledge” Category (5 responses)	#Very informative. #Packed with useful information. #The importance of having critical thinking in a time of uncertainty. #Originally law-abiding leadership should be a topic that is difficult to understand, but the design of the lesson facilitates my understanding of this topic. #I have learned a lot from studying it.
“Thanks, Appreciation and Regards to the Teacher and Assistant” Category (4 responses)	#Thank you. #Thank you. #Teacher prepares the lesson well. #Teacher helped me feel comfortable voicing my opinion about this sensitive topic.
“Law abidance and law abidance Leadership” Category (17 responses)	#Learnt to be law-abiding. #The most important thing I learned is not to try to break the law. #Everybody has the responsibility to obey the law. #Learned much legal knowledge. #Have a deeper understanding of the importance of law abidance. #Help me regulate my behavior through understanding what can or cannot be done. #Law is close to us, and a full and objective understanding of the law is important for the development of both individuals and society. #Reliable content, hopefully, can help me in the future. #I learned a lot of knowledge of the law. #Understand the importance of law to society. #Good to know more about law abidance. #I have learned more about laws. #Be a law-abiding citizen. #Understand more about the concepts of laws. #I have learned more about laws. #It is the duty of every citizen to ensure national stability and not incite racism. #Respect others’ opinions toward different NSL.
“History and Politics Related to National Security” Category (5 responses)	#Have a deeper knowledge of the political system of Hong Kong. #The recent history of China. #Understanding of modern Chinese history. #This lecture enables me to have a deeper understanding of the history of Hong Kong. #Increases my understanding of the history of the country.
“Sense of Belonging to Hong Kong and China” Category (3 responses)	#Increases my passion for the country. #I am quite looking forward to 2047. #I feel the warmth of President Xi.
“Understanding and importance of National Security” Category (5 responses)	#promotes my understanding of national security. #know more about national security. #importance of rules, national security law components. #have known more about national safety law. #Learning clear definitions and details about NSL.
“Hong Kong National Security Law, Security Laws” or NSL Category (47 responses)	#I have gained knowledge of National Security Law in Hong Kong. #Knowing NSL. #Clarify myths about NSL. #This course better my understanding of the NSL. #Importance of NSL. #Penalties of subversion. #Gain a deeper understanding of the National Security Law. #Further understand NSL. #NSL. #deeper understanding of NSL. #I have learnt the importance of NSL and what would be the consequences of violating it. #I have a more complete understanding on the NSL. #I have learned SNL and clarified some myths. #I’ve learned 4 types of crimes and the importance of obeying NSL. #I have learned how to identify different crimes in NSL. #I learned the 4 major crimes and many cases about NSL. #Learn more about NSL. Some cases depend on the situation to determine we violate the law or not. #Before this lecture, I have limited knowledge on NSL. But this lecture does help me to learn more about NSL in Hong Kong, like the term “subversion.” #More understanding of NSL. #More familiar with the NSL. #The lecture content helps me understand NSL. #I know the details of NSL. #The punishments for violating the NSL. #I learned about the consequences of not following NSL. #I like the last part of “myth”; it clarifies some information. #It does help me to clarify some myths related to National Security Law in Hong Kong. #Clarify the reasons for implementing NSL. #Through this lecture, I am able to learn more about NSL in Hong Kong, such as the details of the rules. #The lecture helped me understand NSL. #In this lesson, I learned about the importance of national security laws. #I learned more about the National Security Law (NSL). #Solved some myths about NSL. #Can clearly understand the background of NSL. #National Security Law. #I have learned the difference between reform and revolution and the NSL in Hong Kong. #I know the 4 crimes in NSL. #I learned to distinguish between different offenses in NSL. #This lesson helps us to understand what NSL is. #The lesson let us view it in a comprehensive way; the lesson let us think deeply and decide our position on this law. #Know deeper about NSL. #Understand how NSL can protect the citizen. #This lecture helps me understand more about the National Security Law. #Clear some of my misconceptions about the law. #I learned more about national security law. #Good to know more about National Security Law. #It is good to let students understand the implementation objectives of NSL. Otherwise, we will selectively ignore some knowledge. #It is good to let students know that many countries (including the US and UK) have NSL.
**Neutral/undecided responses (5 responses)**	#If we could get a soft copy of the National security law explanation separately. #This lecture helps us understand NSL, which is remote from us in real life. I hope my classmates can enjoy the lecture. #The strength of a democratic country depends on people; an ideal NSL should attempt to help people. #Russian warship go f yourself. Although this lecture is not interesting to me, it is important for us as we need to be cautious about our behavior and whether we would fall into crime. #Blow up the fish.
**“Negative” responses (6 responses)**	#Useless lecture, wasting my time. #Too much content on introducing and explaining National Security Law. #The lecturer should monitor the chatroom more frequently because he did not know he was muted for like 10 minutes while people in chat kept notifying him. #It is sadly a “Party” Security Law. #Just wish when the lecturer is talking about mainland China, he could refer to it as mainland China and HKSAR, not China and HK. That’s just a little bit confusing. #When the Nazis brought in the Communists, I kept silent; I was not a communist. When they imprisoned the Social Democrats, I kept silent; I was not a Social Democrat. When they brought in the trade unionists, I did not protest; I was not a trade unionist. When they fetched the Jews, I kept silent; I was not a Jew. When they picked me up, there was no one left to protest (translated from a foreign language).

Students responded according to the following instruction: “Please write down your comments about this lecture (e.g., what you have learned in this lecture). If there is no comment, please write ‘NIL”’.

## Discussion

There are two unique features of this study. First, as education on the Hong Kong National Security Law is very new in Hong Kong, there are different views on the related initiatives. Those who support this policy argue that it is legitimate for young people in a country to learn about national security and related law. On the other hand, critics and those who hold skeptical views warn that such education may be “brain-washing” in nature. To demystify the myth that related education is “brain-washing,” we have to collect evidence to understand the views of the students who have taken such courses. Second, under the pandemic, there is a need to understand the effectiveness of different forms of teaching and learning (Shek et al., 2022b). With reference to education on national security, while findings showed that the related education based on the hybrid mode (i.e., simultaneous face-to-face plus online teaching in a classroom) was well-received by students, we have to understand whether the related education was also well-received by students under the online mode. Although there are studies showing that online lectures, including Service-Learning, worked well in Hong Kong (Leung et al., 2021; Lin and Shek, 2021; Shek et al., 2022a), we do not have any research findings on the perceived effectiveness of national security education delivered *via* online mode.

We have several observations regarding the quantitative findings. First, confirmatory factor analyses showed that there are three dimensions intrinsic to the 26-item scale assessing the perceptions of the students, and the factors are stable across the two random subsamples. The present findings provide support for the factorial validity of the measure. Reliability analyses also showed that the total scale and subscales were reliable; there is also support for the convergent and discriminant validity of the measure. In response to the call for developing more outcome measurements in the Chinese context (Shek et al., 2021b), this study contributes to the Chinese scientific literature. Practically speaking, the present study suggests that this measure can be objectively used to assess national security education in future. As research on national security education is sparse in the scientific literature, the present study contributes to the field.

Second, the present findings showed that the responses of the participants are generally positive in nature. Regarding lecture attributes, students had positive views of the design and content of the lecture. Besides, they also perceived the lecture process to be positive, with good interaction amongst the students as well as high teacher-student interaction. Furthermore, they perceived many benefits of the lecture, including the opportunity for reflection, promotion of critical thinking, interpersonal communication, and understanding of successful leader attributes. For the teacher attributes, teachers were perceived as knowledgeable in the subject area, able to promote mutual respect during class, using different methods to promote student learning, and able to take care of all students. Most importantly, students agreed that the lecture was able to promote their understanding of law abidance, law abidance leadership, national security, and the Hong Kong National Security Law. In particular, students agreed that they would try their best to be responsible citizens and law-abiding leaders. These findings are generally in line with the findings reported by Shek et al. (2022b). Taken together, the available evidence demystifies the myth that students are resentful of national security education. Of course, it is noteworthy that although 1,312 students took this subject, 914 students (69.7%) responded to the post-lecture evaluation. Nevertheless, it is noteworthy that the response rate was higher than in other studies conducted in Hong Kong (59% response rate reported in Ng, 2022) and the recommended response rate of 35% if the student number is greater than 50 (Nulty, 2008). Of course, whether the participants can represent all students registered for this course is a question for further consideration. However, even if we assume that those who did not respond held negative views on law abidance leadership, the findings still suggest that a majority of the students were positive about this lecture. Of course, under this assumption, we have to understand the views of around one-third of students who did not have positive perceptions of NSL.

Third, we identified several predictors of overall satisfaction with the 3-h lecture on law abidance leadership. Basically, lecture attributes, teacher attributes, and appreciation of law abidance leadership were significant predictors of overall satisfaction. However, amongst the three predictors, teacher attributes exerted the weakest effect. Actually, this is not a novel finding. In previous studies examining factors predicting overall satisfaction with a program, teacher attributes were also identified as a weak or non-significant predictor of overall satisfaction with a program (Shek and Sun, 2014; Sun and Shek, 2014; Shek et al., 2017a; Sun and Shek, 2014). One possible explanation is that the ceiling effect may account for this observation—because ratings for teachers are generally high, the lack of variation in the ratings may contribute to this observation. In contrast, appreciation of law abidance leadership showed the strongest effect on overall satisfaction with the lecture. This observation suggests that the promotion of students’ understanding of law abidance, law abidance leadership, national security, and Hong Kong National Security Law, as well as their determination to be law-abiding, is important in shaping students’ satisfaction. These observations help to develop theoretical models on the predictors of student satisfaction with law abidance leadership programs (i.e., helping students to understand the related knowledge and commit to law abidance leadership).

Finally, the qualitative findings are generally encouraging, with most of the responses coded positive, with a few negative, neutral, or undecided responses. This observation is in line with the findings reported by Shek et al. (2022b). Again, as only around 70% of the students joined the post-lecture evaluation, interpretations of the findings should be cautious. In the extreme case, one might counter-argue that 30% of the students might hold negative views, although the majority of responses are still positive in nature. Hence, there is a need to understand why around 30% of the students did not join the post-lecture evaluation.

Law abidance leadership is important for university students for several reasons. First, following the requirement of the law reinforces respect for the law. In fact, it takes time to build up a law abidance culture. Second, justice is promoted when one follows the law. Again, this is a matter of cultivating a culture promoting justice. Third, leaders will serve as role models for their subordinates when they observe the requirement of the law. Obviously, we have to ask how we can promote law abidance leadership. Besides enriching students’ knowledge in this area, we have to realize the foundational skills underlying law abidance, such as cognitive competence, social competence, and moral competence. In view of the weak life skills education in Hong Kong, there is a need to reflect on how such foundational soft skills can be strengthened (Shek et al., 2021a).

From a replication perspective, the present findings replicated the findings reported by Shek et al. (2022b) in a new sample of students taking the subject *via* another mode (i.e., online teaching and learning mode) at another time. Replication has been regarded as a crisis in social sciences. McShane et al. (2019) commented that “the validity of research in the biomedical and social sciences is under intense scrutiny at present, with published findings failing to replicate at an alarming rate. This problem appears particularly acute in psychology, where the failure to replicate several prominent findings” (p. 99). Makel et al. (2012) also pointed out that the effort to replicate studies in Psychology was very low, with 1.6% of the studies in Psychology utilized “replication” in the text of the publications (6). Besides, Stevens (2017) asserted that “Though science depends on replication, replication studies are rather rare due to an emphasis on novelty: journal editors and reviewers value replication studies less than original research” (p. 1). Fecher et al. (2016) also pointed out that although a replication crisis exists in many disciplines and researchers generally endorsed the importance of replication, researchers usually counted on other researchers to replicate scientific findings (Burman et al., 2010). Hence, the present study constitutes a modest attempt to replicate the previous findings in the area of national security education.

Finally, as law abidance leadership education with respect to NSL is politically sensitive, there is a need to take several local and international contextual factors into account when interpreting the findings of this study. First, students in Hong Kong may not have a good understanding of the law and the importance of abidance. Kennedy and Chow (2009) conducted a survey on attitudes to law and law-related issues among young people in Hong Kong They found that young people in Hong Kong did not have a sophisticated conceptual understanding of law requirements when it came to law and personal values. Findings also showed that students were prepared not to follow the law if they considered the law was unjust, although they regarded disobeying such laws as bad for democracy. A recent survey found that 27.2% of the surveyed young people in Hong Kong would not follow laws if they regarded the laws as unreasonable (Hong Kong Federation of Youth Groups, 2021). These findings suggest that there is a need to help young people understand the law as well as to respect the need for law abidance.

Second, with reference to Hong Kong NSL, there are contrasting views on its legitimacy and impacts. According to Basic Law, Hong Kong has a constitutional responsibility to enact its own laws on safeguarding national security (Article 23), but it is not implemented in the past 25 years. To Beijing, without a national security law, Hong Kong is a major weakness in the nation’s overall security, and it directly impacts residents, as stated by Chinese officials (South China Morning Post, 2018). The anti-extradition protests in 2019 were not only a challenge to the legitimacy of the Central and Hong Kong governments but also construed as an attempt at initiating a “color revolution” which raised Beijing’s deep concern about undermining its national security and turning into a broader anti-China movement (Lo, 2021). In response, the Central Government enacted the Hong Kong NSL in late June 2020 to restore public order in Hong Kong.

Third, perceptions of the Hong Kong NSL are polarized in both local and international contexts. Although the Chinese authorities and the government of Hong Kong described NSL as a key to Hong Kong’s long-term prosperity and stability (Lau, 2021), there are both local and international critics commenting that the law was problematic in the definition of offenses as well as scope and cracking the firewall between Hong Kong and mainland China’s legal systems. While the pro-democracy and western countries suspect that the Hong Kong NSL would undermine the autonomy of the HKSAR and exert controls over society, which cause serious challenges to civil and political rights (Clift, 2020), supporters of the Central and Hong Kong governments advocate the law as a mean to cease the social unrest and restore social stability. The implementation of the Hong Kong NSL has sparked international criticisms. Some western countries view the NSL as a sign that the central government is having increasing interference in Hong Kong’s internal affairs, which is not in conformity with international commitment intrinsic to the Sino-British Joint Declaration of 1984 (European Parliament, 2020). Sanctions were imposed against officials of mainland China and Hong Kong, and political asylum or special arrangements were granted to some of the protest leaders after the enactment of the Hong Kong NSL by some western countries (Young, 2021). At the same time, 53 countries signed a statement at the United Nations supporting the Hong Kong NSL.

Fourth, people do use different standards to understand political events. For example, while vandalization of government buildings (e.g., Hong Kong Legislative Council building) or blockade of major roads in Hong Kong was considered by some westerners a legitimate reaction to the repression of authoritarian control, the Capitol Building incident in 2021 and the Freedom Convoy in Ottawa in 2022 were described as “an attack on democracy.” Besides, while more people perceived the events as crimes or even terrorism affecting national security, some other people considered protesters’ acts ways to overturn the election results or government policies. Though laws related to national security are common in many western countries for years, the controversy on the topic of national security still exist.

Fifth, our lecture aims to provide background and factual information on Hong Kong NSL and invite students to reflect on the necessity of law abidance in society. Therefore, the 3-h lecture does not merely include the Hong Kong NSL *per se*. Instead, it consists of five parts aiming to provide students with comprehensive information—law-abiding leadership (e.g., the reasons why a leader should obey the law), a brief overview of modern Chinese history (“Century of Humiliation”), concepts of national security and national security law, the Hong Kong National Security Law, and issues surrounding the Hong Kong National Security Law. For example, discussion on the necessity of law abidance in a society, court cases, and reasons for verdicts are used in the lecture to provide knowledge and stimulate reflection. We also discuss whether NSL restricts freedom of speech in Hong Kong. Our basic stance is that students should understand the NSL so that they will not step on the “red line.” As teachers, while we respect the personal choice of the students, we strongly encourage and admonish students to follow NSL and reflect on the various issues related to it.

Sixth, we conducted the evaluation *via* a student feedback survey, which is a mainstream evaluation approach in higher education. As students’ learning experience is expected to influence learning outcomes (Palmer and Holt, 2009), examining their satisfaction is an important aspect of understanding the effectiveness of NSL education. In fact, studies on student feedback survey are very popular in leading education journals, including *Frontiers in Education* (e.g., Cannon et al., 2022). In this study, we strived to examine the effectiveness of NSL education for university students *via* a scientific approach (i.e., student feedback survey) instead of a political approach (i.e., attitudes to NSL). We covered the basic facts of NSL (e.g., NSL in other countries and the importance of national security) and examined its effectiveness without taking sides. In fact, some qualitative findings are not entirely positive, which provides a good pointer for the refinement of the pedagogy.

Finally, understanding people’s views toward controversial issues, such as political issues, in a scientific way, is not uncommon in the field of education. In some isolated attempts, researchers have conducted studies on understanding students’ views about teaching the Northern Ireland conflicts. Barton and McCully (2005) conducted interviews with students and found that students selectively chose to learn information from the required national curriculum of Northern Ireland Conflicts history matched with their political or religious communities. Barton and McCully (2012) further suggested that the related curriculum should include explaining the history based on multiple historical perspectives so that it can address the affective components of students when learning controversial history. Obviously, teaching sensitive and controversial topics is not easy, and there are many issues faced by teachers (Lowe and Jones, 2010). However, as commented by Heath et al. (2017), “addressing sensitive content is a professional responsibility for teachers, disciplines, and universities” (p. 5). Obviously, evaluation and replication of evaluation findings play an important role in shaping the pedagogies and teaching processes involved when teachers teach sensitive topics in their classes.

There are several limitations of the study. First, as the students joined the study in an anonymous manner, we should be cautious about generalizability of the findings. Second, as there is no comparison group in this study, it would be helpful to include a comparsion group to understand the program effect. Nevertheless, it should be noted that student feedback survey is commonly used in the higher education. Finally, it would be helpful to include other evaluation mechanisms, such as qualitative evaluation and evaluation based on different stakeholder. In fact, we have collected data from teachers (Shek et al., 2022c; Shek et al., in press) and conducted a focus group study. The findings arising from these studies converged to show that the students’ learning experience was positive and there are perceived benefits of this program for the students. Of course, as these studies are initial attempts to understand the impact of law abidance leadership education program, these studies should be replicated in future.

## Conclusion

This study examined students’ perception of a law abidance leadership program based on the post-lecture evaluation data collected with 914 students. The results revealed that students were generally satisfied with the lecture attributes and agreed that the lecture helped them understand the importance and value of law abidance and national security. The present findings replicated the findings of a previous study by Shek et al. (2022b) and underscored the effectiveness of national security education.

## Data availability statement

The raw data supporting the conclusions of this article will be made available by the authors, without undue reservation.

## Ethics statement

The studies involving human participants were reviewed and approved by the Institutional Review Board (or its Delegate) at The Hong Kong Polytechnic University. The patients/participants provided their written informed consent to participate in this study.

## Author contributions

DS: conceptualization, supervision, and funding acquisition. DS and DD: methodology and formal analysis, and writing—original draft preparation, review, and editing. DS, DD, XZ, and XL: investigation and project administration. DD: data curation. All authors have read and agreed to the published version of the manuscript.
